# Intracycle power and ventilation mode as potential contributors to ventilator-induced lung injury

**DOI:** 10.1186/s40635-021-00420-9

**Published:** 2021-11-01

**Authors:** John J. Marini, Philip S. Crooke, Pierre Tawfik, Robert L. Chatburn, David J. Dries, Luciano Gattinoni

**Affiliations:** 1 Pulmonary and Critical Care Medicine, University of Minnesota and Regions Hospital, Minneapolis/St. Paul, MN 55101 USA; 2 Department of Mathematics, Vanderbilt University, Nashville, TN USA; 3 Department of Medicine and Respiratory Institute, Cleveland Clinic, Cleveland, OH USA; 4 Department of Anesthesiology, Intensive Care and Emergency Medicine, Medical University of Göttingen, Göttingen, Germany; 5 Regions Hospital, MS-11203B, 640 Jackson St, St. Paul, MN 55101 USA

**Keywords:** Mechanical ventilation, Mathematical model, Ventilator-induced lung injury, VILI, Power, Intracycle power, Energetics, Modes of ventilation

## Abstract

**Background:**

High rates of inflation energy delivery coupled with transpulmonary tidal pressures of sufficient magnitude may augment the risk of damage to vulnerable, stress-focused units within a mechanically heterogeneous lung. Apart from flow amplitude, the clinician-selected flow *waveform*, a relatively neglected dimension of inflation power, may distribute inflation energy of each inflation cycle non-uniformly among alveoli with different mechanical properties over the domains of time and space. In this initial step in modeling intracycle power distribution, our primary objective was to develop a mathematical model of global *intracycle* inflation power that uses clinician-measurable inputs to allow comparisons of instantaneous ICP profiles among the flow modes commonly encountered in clinical practice: constant, linearly decelerating, exponentially decelerating (pressure control), and spontaneous (sinusoidal).

**Methods:**

We first tested the predictions of our mathematical model of passive inflation with the actual physical performance of a mechanical ventilator–lung system that simulated ventilation to three types of patients: normal, severe ARDS, and severe airflow obstruction. After verification, model predictions were then generated for 5000 ‘virtual ARDS patients’. Holding constant the tidal volume and inflation time between modes, the validated model then varied the flow profile and quantitated the resulting intensity and timing of potentially damaging ‘elastic’ energy and *intracycle* power (pressure–flow product) developed in response to random combinations of machine settings and severity levels for ARDS.

**Results:**

Our modeling indicates that while the varied flow patterns ultimately deliver similar total amounts of alveolar energy during each breath, they differ profoundly regarding the potentially damaging pattern with which that energy distributes over time during inflation. Pressure control imposed relatively high maximal intracycle power.

**Conclusions:**

Flow amplitude and waveform may be relatively neglected and modifiable determinants of VILI risk when ventilating ARDS.

**Supplementary Information:**

The online version contains supplementary material available at 10.1186/s40635-021-00420-9.

## Background

The mechanical stimulus for ventilator-induced lung injury (VILI) is currently understood to involve repetition of excessive tissue strains at the alveolar level. Excessive strain per inflation cycle may either physically deform vulnerable structural elements or serve a signaling function for inflammation [1–3]. Incremental strain during inflation requires the delivery of energy, quantified as the product of transpulmonary pressure and the resulting volume change [4]. Because contiguous lung units expand at different rates, global stress may not be evenly shared within the parenchyma, especially by pre-injured tissue with heightened viscoelastance [5–7]. Therefore, when coupled to transpulmonary tidal pressures of sufficient magnitude, faster transfer of the energy delivered during the breath may augment the risk of damage within a mechanically heterogeneous lung (e.g., ARDS). Apart from flow amplitude, the clinician-selected flow *profile* (waveform of gas delivery) may distribute the total inflation energy non-uniformly over time.

Power is the energy delivered per unit time of any duration. When defined as cumulative inflation energy applied to the entire lung per minute, all pressure components of power have the potential to contribute to VILI [4]. Although strain rate has been shown experimentally to be potentially important to VILI risk [8], the rate of energy transfer during the inflation cycle itself—the *intracycle* power (ICP, an instantaneous product of pressure and flow)—has only recently been defined for the common flow waveforms applied to the intubated patient (e.g., constant, linearly or exponentially decelerating) [9].

We reasoned that by influencing incremental strain rate, the airflow waveform may influence the actual micro-strains experienced by vulnerable fibrils of the matrix that are subject to greater stress focusing [10, 11]. The mechanical factors that influence damaging micro-strains are complex and undoubtedly vary across local stress environments. Yet, all depend on energy input during inflation, and the only measurable variables that relate to energy and power which can be quantitated at the bedside are airway pressure and flow—the determinants of global intracycle power. Ultimately, a refined and granular mathematical model will incorporate multiple interacting compartments that takes account of non-linear relationships between time and regional pressure and volume that as yet cannot be clinically estimated. Reaching our ultimate goal of incorporating such complexity will require a progressive series of more elementary ‘building blocks’. To serve as an essential first step in this conceptual framework, we developed a comprehensive but highly simplified one-compartment mathematical model of the intracycle power applied to the lungs’ entirety. This model compares clinically relevant flow waveform options regarding their delivery of global instantaneous power when applied to lung of varying resistance and elastance properties, as assessed by the parameters already available to the bedside clinician. In theory, the instantaneous power profiles of different flow waveforms may influence strain levels encountered in varied mechanical environments. (However, in this one-compartment model, we did not attempt to predict how the power would distribute *spatially* among the varied units comprising a mechanically heterogeneous lung.) To verify that our mathematical model faithfully imitates the ‘real world’ environment, we then confirmed model predictions for overall ICP with a patient ventilator that drove a physical simulator of the respiratory system programmed with a broad range of the lumped, constant, and global mechanical parameters (resistance, *R*, and compliance, *C*) typically encountered in ICU practice. After verification of its predictive accuracy in a physical environment, we used our mathematical model to compare the performance of different flow waveforms for simulated disease settings and typical clinician choices that influence inflation energy (tidal volume, PEEP and ventilation frequency). Our observations suggest that in addition to the flow amplitude, the flow waveform may a be relatively neglected and modifiable determinant of VILI risk.

## Methods

### Mathematical model of volumes

A mathematical model was developed to simulate ventilation into and out of a simplified one-compartment respiratory system. The details of this model for intracycle power and its subcomponents are elaborated in the SUPPLEMENT [Additional File] sections E1 and E2.

[Note: These additional files are also made available separately and simultaneously on the following public data repository.]

https://tinyurl.com/xhx6u9md

https://figshare.com/articles/journal_contribution/Marini_Integrated_Supplement_docx/14555556

https://figshare.com/articles/figure/Marini_Figure_E1_Total_and_Elastic_Power_ARDS_vs_COPD_pdf/14555595

In broad summary outline, the total duration of each breath (*t*_tot_) was subdivided into an inspiratory phase ($0\le t\le {t}_{i}$) and an expiratory phase (${t}_{i}\le t\le {t}_{\text{tot}}$). We assumed ‘lumped’ but clinically measurable mechanical characteristics for resistance (*R*) and compliance (*C*).

The “equations of motion” built upon this base assume that the applied pressure at the airway opening is balanced by pressures developing in response to resistance and elastance (inverse of compliance) as well as by the residual alveolar pressure at end-expiration, defined as $P_{{{\text{residual}}}} = P_{{{\text{ex}}}} = {\text{total}}\,{\text{PEEP,}}\,{\text{ the}}\,{\text{ sum }}\,{\text{of }}\,{\text{PEEP }}\,{\text{and }}\,{\text{auto}}\,{\text{PEEP}}$. Thus:

$$P_{{{\text{resistive}}}} + P_{{{\text{elastic}}}} + P_{{{\text{residual}}}} = P_{{{\text{applied}}}} .$$

These assumptions lead to the following mathematical model that comprised a coupled pair of initial-value problems for the unknown volume functions for passive inspiration and expiration${V}_{i}\left(t\right)\mathrm{ and }\,{V}_{e}\left(t\right),\mathrm{ respectively}$. At a given time *t*:

Inspiration (i):

$$R\frac{{{\text{d}}V_{i} }}{{{\text{d}}t}} + \frac{{V_{i} }}{C} + P_{{{\text{ex}}}} = P_{{{\text{aw}}}} ,\quad 0 \le t \le t_{i}$$

$$V_{i} \left( 0 \right) = 0$$

*Expiration (e):

$$R\frac{{{\text{d}}V_{e} }}{{{\text{d}}t}} + \frac{{V_{e} }}{C} + P_{{{\text{ex}}}} = {\text{PEEP}}, \quad t_{i} \le t \le t_{{{\text{tot}}}}$$

$$V_{e} \left( {t_{i} } \right) = V_{i} (t_{i} ) = V_{T.}$$

*(During expiration, flow is assumed to be a negative quantity.)

Each differential equation in this generalized mathematical model can be solved for ${V}_{i}\left(t\right)\mathrm{ and }{V}_{e}(t)$. Our simplified model emulates ventilation-related variables that result from different ventilatory patterns (flow waveforms). In addition to the sinusoidal flow pattern of normal spontaneous breathing (SF), we studied model predictions for three popular modes of ventilation: (1) constant inflation pressure (exponentially decelerating flow) ventilation (*P*_set_)$;$ (2) constant flow ventilation (CF); and (3) linearly decelerating flow ventilation (DF).

The total *intracycle* *power* (*ICP*_*T*_) is the sum of the values at inspiratory time *t* for the power components corresponding to flow-resistive and elastic power components (Additional file 1: section E2):

$${\text{ICP}}_{T} = \left[ {R\frac{{{\text{d}}V_{i} }}{{{\text{d}}t}}\left( t \right) + \left( \frac{1}{C} \right)V_{i} \left( t \right) + P_{{{\text{ex}}}} } \right]\frac{{{\text{d}}V_{i} }}{{{\text{d}}t}}\left( t \right).$$

Physical validation of this general mathematical model was conducted as described below.

#### Intracycle power above alveolar pressure threshold for damage

**Fig. 1:**
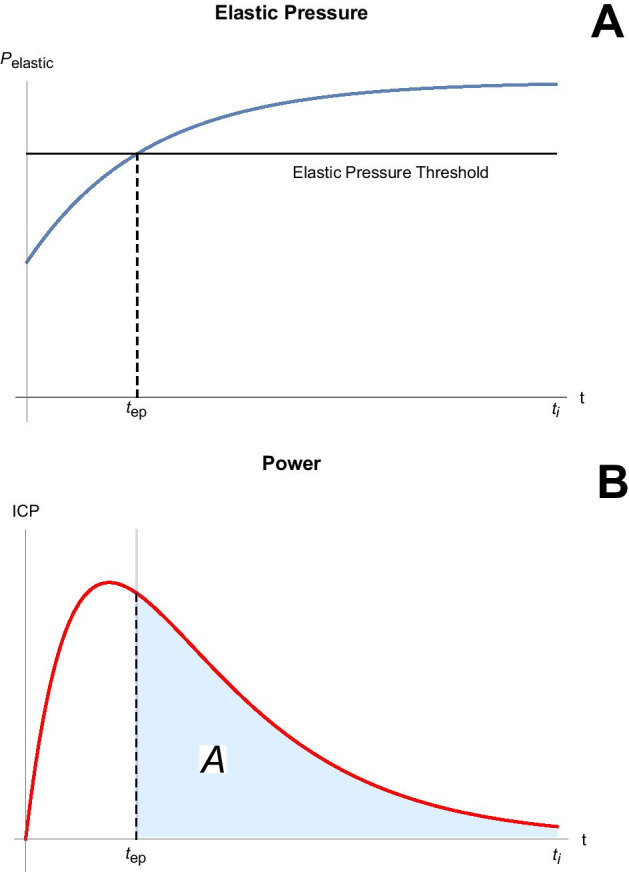
Schematic depiction of elastic pressure (**a**) and intracycle power (**b**) as functions of inspiratory time (*t*_*i*_) for the Pset flow profile at PEEP = 0 cmH_2_O. Note that the concept illustrated here in generic form applies equally well to total intracycle power and to its driving elastic and dynamic elastic components. As the lung inflates, elastic pressure reaches a pressure threshold at time *t*_*ep*_ beyond which further increments of power have potential to contribute to damage. Although PEEP is assumed to be zero in this illustration for clarity, identical principles apply when PEEP is added

Focusing on a logical VILI culprit for the individual tidal cycle, intracycle power, we reasoned that not every such power level associates with damaging stress/strain; sufficient alveolar pressure (reflecting stress) as well as sufficient power must both be applied simultaneously. In other words, a necessary condition for damaging power is that the alveolar pressure level (${P}_{elastic}(t)=\frac{{V}_{i}\left(t\right)}{C}+{P}_{ex}$) must exceed some prescribed threshold. Therefore, we examined the intracycle power function, $ICP(t)$, for those times after the elastic pressure function crossed its designated threshold (Fig. 1, further explained in Additional file 1: Section E6).

The area enclosed by intracycle power vs. time beyond the alveolar pressure threshold reflects ‘potentially damaging energy’, but does not identify the level nor the time after pressure threshold crossing, ${t}_{ep}\le t\le {t}_{i}$, when intracycle power assumes instantaneously large values. For this reason, we identified the highest amplitude of the intracycle power function during the period after the elastic pressure function crossed its designated threshold. This maximum intracycle power value that occurs above the pressure threshold (*M*) and the ‘above threshold’ energy area (*A*) are two potential energetic indicators of damage risk. Using them, we calculated ‘damage risk’ indicators for each of 5,000 ‘virtual’ patients with ARDS of varying severity, each ventilated with all four flow waveforms (${P}_{set}$, CF, DF, and SF), and each primed by disease-relevant randomized input variables (details in Additional file 1: sections E4, E5 and Table E1). Although we focus in this communication on ARDS, a condition predisposed to VILI, we followed the same ‘sampling’ procedure for normal and severely obstructed virtual patients, 5000 in each category, as also detailed in the Supplement. We note here that the arbitrarily set elastic (‘alveolar’) pressure threshold of 20 cmH_2_O was crossed in 4.5% of normal, 31.2% of severely obstructed, and 72.0% of ARDS samples.

### Physical validation of the mathematical model

**Fig. 2:**
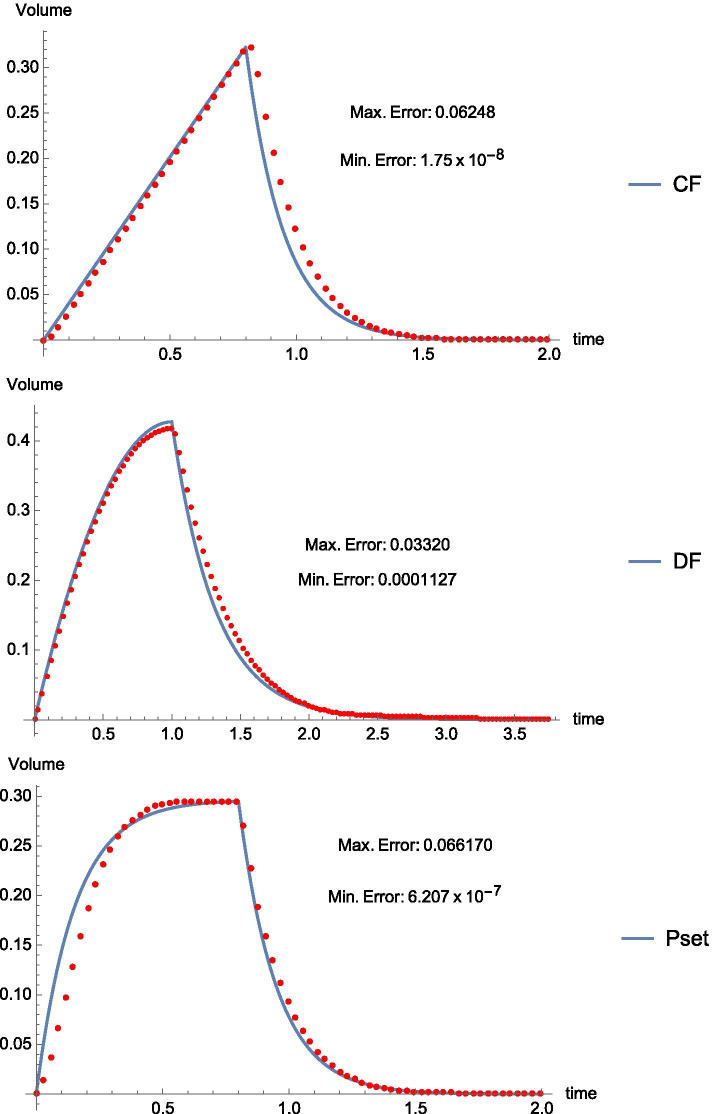
Alveolar volume (L) versus time (s) for three flow profiles applied to lungs with same tidal volume, inspiratory time, and mechanical properties for R and C (PEEP = 0 cmH_2_O). Mathematically predicted (solid line) and physically observed (dotted line) quantities agreed particularly closely for the inflation phase of the flow-controlled modes (constant [CF] and decelerating [DF]). Inflation by the mechanical simulator lagged slightly in response to *P*_set_ inflation, due to the ventilator’s ‘ramped’ early pressurization algorithm

We verified the ‘real world’ relevance of predictions of our mathematical model by driving with a commercial ventilator a servo-actuated lung simulator (IngMar ASL-5000^©^) that allows priming with the relevant resistance and compliance inputs as well as direct measurement of all outputs actually observed in this physical system. To test the accuracy of the mathematical model’s predictions over a wide, clinically relevant range, we varied the mechanical properties of the simulator to reflect passive inflation of three prototypical intubated patient types: severe acute respiratory distress syndrome (ARDS), severe airflow obstruction (e.g., COPD), and normal lungs. (The mathematically modeled sinusoidal flow pattern of unassisted spontaneous breathing is not currently offered by the ventilator used.) For each simulated ‘patient’, exponentially decelerating (${P}_{set}$), linearly decelerating (DF, using three slopes) and constant flow (CF) waveforms were applied by a commercially available ventilator (Maquet Servo-U^©^) tasked to deliver identical tidal volumes and inspiratory times for each flow waveform. Overall, 42 such experiments were performed with patient parameters, ventilator settings and modes representative of ARDS, COPD, and normal lung (Additional file 1: Table E2). We made two types of comparisons between the predictions of the mathematical model and observed mechanical model outputs: (1) plotting of predicted lung volume, $V(t)$, during inspiration and expiration against the discrete volume values produced by the mechanical lung; (2) point-by-point comparison of the model and mechanical lung, assessing the maximum absolute difference over the whole breath (Fig. 2). All computations and statistical analyses were performed using *Mathematica*™ (Wolfram Research, Champaign IL, USA).

## Results

### Correspondence of mathematical and physical models

A tight correlation between mathematical and physical models held for virtually all tested variations of mechanical properties, inflation mode and settings. In general, the mathematical model and mechanical simulator closely agreed, with absolute errors of < 1% during inspiration for ventilator modes CF and DF and marginally more with P*set*. Comparative data, including exceptions and explanations are detailed in the on-line Supplement, section E3.

### ARDS model predictions: comparison of flow waveforms regarding damaging potential

The 5,000 virtual experiments simulating ventilation with randomized ARDS inputs provided evidence that intracycle total power area above pressure threshold ($A,\text{ an }\,energy\,\text{ measure}$) and the maximum intracycle power value exceeding the pressure threshold (*M*, a measure of instantaneous *power* with damaging potential) may each help indicate which flow pattern carries the least hazard risk for the same average flow rate. These hypothetical damage indicators, as well as their power subdivisions are displayed in Table 1.

**Table 1:** Means and standard deviations of areas and maximum power values that exceed the elastic pressure threshold of 20 cmH_2_O for the 5000 virtual ARDS patient population using the four modes (flow profiles) of ventilation

Statistic/mode	Pset	CF	DF	SF
Driving power area (mean)	0.1698	0.1698	0.1698	0.1698
Elastic power area (mean)	0.4078	0.4078	0.4078	0.4078
Total power area (mean)	0.5128	0.4693	0.4753	0.4849
Driving power area (standard deviation)	0.1968	0.1968	0.1968	0.1968
Elastic power area (standard deviation)	0.3986	0.3986	0.3986	0.3986
Total power area (standard deviation)	0.5376	0.4566	0.4709	0.4751
Driving power maximum (mean)	0.5505	0.4302	0.3215	0.4299
Elastic power maximum (mean)	1.4652	0.8141	0.8202	0.9662
Total power maximum (mean)	2.5949	0.9262	1.0533	1.2000
Driving power maximum (standard deviation)	0.8253	0.4268	0.3301	0.4371
Elastic power maximum (standard deviation)	1.6642	0.6431	0.6687	0.7715
Total power maximum (standard deviation)	3.8029	0.7323	0.9100	0.9800

Driving power above threshold and the PEEP-inclusive elastic power above threshold are subcomponents of the total power exceeding threshold. Note that in contrast to their power maximum counterparts, the driving and elastic power (energy) *areas* do not vary as functions of the flow profile. Power areas are expressed in joules, and maximum power values are expressed in watts

Note that if higher values of total power areas and extremes of power above the pressure threshold are linked with VILI risk, then CF and DF appear preferable over ${P}_{set}$ and SF modes of ventilation. We examined binary statistical comparisons between the ventilation modes for *A*, for *M*, and for their subcomponents. As detailed in the Additional file 1: sections E5 and E6, purely ‘elastic’ energy areas do not vary with flow contour and therefore do not distinguish among flow profiles. When evaluated by the Mann–Whitney test, however, maximal intracycle power values occurring above the pressure threshold (*M*) and each of its power subcomponents discriminated between flow modes far more sharply than did those of the total energy delivered ‘above pressure threshold’, i.e., the damage risk area, *A*. (Table 1 and Additional file 1: sections E6 & E7).

**Fig. 3:**
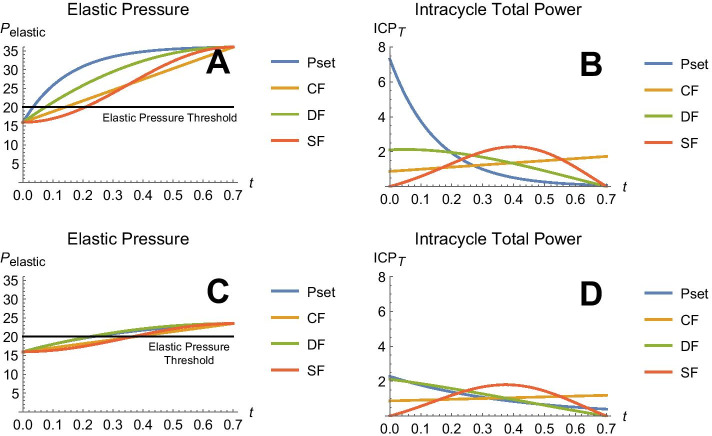
Mathematical model predictions of elastic pressure (*P*_elastic_, cmH_2_O) and intracycle total power (ICP_*T*_, watts) versus inflation time (*t)* in response to each of four flow profiles applied in ARDS: pressure control, *P*_set_; constant flow, CF; decelerating flow DF; and sinusoidal flow, SF. Severe ARDS (panels A and B) is compared with mild ARDS (panels C and D). Tidal volume, inspiratory time and flow resistance settings were identical for each profile

To evaluate the specific influence of abnormal lung mechanics on intracycle power, we also examined predictions for the intracycle total power profiles for mild and severe ARDS using four clinically available flow waveforms of ventilation. Ventilator settings (${t}_{i}=0.7$, ${t}_{tot}=4.0$, ${V}_{T}=0.3 L$, $PEEP=16 {\text{cmH2O}}$) were kept constant for each waveform, along with ‘virtual patient’ resistance ($R=10\frac{cmH2O}{L}/s$). Here, only the compliance was changed: for mild ARDS, $C=0.040$ *L/cmH*_*2*_*O* and for severe ARDS, *C* = 0.015 *L/cmH*_*2*_*O*. The elastic pressure curves for each mode of ventilation are shown in panels A and C of Fig. 3. The corresponding intracycle total power curves are shown in panels B and D. The numerical values for intracycle total power area and the maximum values for three intracycle power functions that correspond to total, driving, and elastic pressures are tabulated in Table 2.

**Table 2:** Mode comparisons of intracycle total power areas (A, joules) and maximum intracycle power levels (M, watts) that are inscribed above a pressure threshold of 20 cmH_2_O for two severities of ARDS

ARDS type	Intracycle power descriptor	Constant pressure	Constant flow	Decelerating flow	Sinusoidal flow
Severe	Area (*A*)	0.8686	0.7748	0.7947	0.8080
	Maximal power (*M*)	5.8591	1.7265	2.2111	2.2833
	*M*_D_	1.0191	0.8571	0.6598	0.8745
	*M*_E_	3.2380	1.5429	1.5935	1.8625
Mild	Area (*A*)	0.3513	0.3547	0.3594	0.3773
	Maximal power (*M*)	1.2739	1.1909	1.5145	1.7969
	*M*_D_	0.2060	0.3214	0.2474	0.3279
	*M*_E_	1.0259	1.0072	1.1714	1.3527

Maximal instantaneous power values are also displayed for the driving (*M*_D_) and elastic (*M*_E_) subcomponents of total power

It is clear from Fig. 3 that the elastic pressures are higher and the durations after crossing the elastic pressure threshold are longer in severe ARDS as opposed to mild ARDS. In turn, these attributes affect the ‘above pressure threshold’ areas of intracycle power (i.e., potentially damaging energy) as well as the maximum values of intracycle power exceeding the pressure threshold (Table 2).

Our primary findings can be summarized:

- Interactions among mechanical characteristics and flow waveform may affect key measures of total intracycle power and its and subcomponents relevant to VILI.
- The ‘above pressure threshold’ magnitudes of maximum instantaneous elastic power (*M*) and of energy delivered above pressure threshold (*A*) were influenced by simulated disease severity, by flow waveform and intuitively, by end-expiratory residual pressure (the latter not shown). The *P*_set_ mode produced higher mean values of these VILI-related energetics variables than did the flow-controlled modes, and with greater variability.

## Discussion

Our modeling indicates that while the varied flow patterns encountered in clinical practice ultimately deliver similar total amounts of mechanical energy to the alveoli (‘elastic’ energy) during a single inflation with a given fixed *average* amplitude of flow, they differ profoundly regarding the pace and pattern with which they distribute that elastic inflation energy over the inflation time and regarding the maximum amplitude of intracycle power they generate. We also found that if one specifies a minimum threshold level of alveolar (elastic) pressure at which VILI injury becomes a concern, varying the flow waveform will modify the *potentially* *damaging* maximum instantaneous intracycle power and perhaps the suprathreshold elastic energy. Note that importantly, the assigned level of the alveolar pressure threshold influences the magnitudes of ‘above threshold’ energy and maximum power, but does not influence the hierarchy among flow waveforms. Finally, our analysis demonstrates that estimates of total intracycle power determined by airway pressure and flow measured within the ventilator’s external circuit may differ impressively from *alveolar* (‘elastic’) intracycle power, depending on the flow waveform and the mechanical properties of the inflating lung. It is important to emphasize that once total energy per cycle is determined from measurable flow and circuit airway pressure, the relevance of flow amplitude and pattern to damage is conditioned by its capacity to augment (or dissipate) strain in micro-zones with heterogeneous viscoelastic properties and heightened stress focusing [5, 6]. By implication, the externally measurable total energy per cycle, though a key determinant of ‘power’ assessed per minute [3, 4, 12], may not fully characterize the hazard imposed at the alveolar level by its associated intracycle power.

In a recent publication, we called attention to the theoretical importance of the flow waveform to the energetics of VILI causation [9]. In this present work, we detail the implications of the equations that define the dynamics of intracycle energy application and verify their relevance to the performance of a ‘real world’ mechanical simulation of the ventilator–respiratory system that tracks all clinician-measurable variables determining elastic (alveolar) and total power. Although as yet untested in biological experiments and clinical settings, tight correspondence maintained across the wide range of modes and inputs to these mathematical and mechanical models suggests that similar principles and relationships may apply in ICU practice, as well.

When defined as the product of total per-cycle energy and ventilating frequency, ‘power’ is a valid but *cumulative* measure of energy load over the fixed interval of one minute [3, 12]. While ventilating frequency and total energy delivered per minute are of demonstrated worth [13], injury hazard must also relate to excessive tissue micro-strains experienced within the span of each tidal cycle. As illustrated by our model, that moment-by-moment energy-loading pattern—power delivered on a short time scale—can be estimated for the entire respiratory system from knowledge of the flow waveform, the targeted tidal volume, the elapsed time from inflation onset, the end-expiratory pressure and the pressure components of inflation that are influenced by resistance and compliance. Our analysis indicates how dissimilar flow waveforms distribute a fixed amount of alveolar elastic energy differently as the lung inflates. In so doing, various waveforms develop maximum elastic power values that may have greater tendencies to avoid or impose hazardous strain. For example, in severe ARDS, the suprathreshold level for elastic intracycle power is crossed earlier and rises higher with the exponentially decelerating flow of *P*_set_ than with the constant flow waveform (Fig. 3).

One might envision that rapid expansion would amplify incremental strains experienced at junctional interfaces between rapidly expanding and sluggish or immobile units [5–7]. A typical value of maximum tidal elastic pressure (‘plateau’) that raises clinical concern is 25–30 cmH_2_O [2]. Yet, because this value represents a global average, in all likelihood, lower threshold pressures (such as the 20 cmH_2_O we illustrated) may be relevant to the zones with the most jeopardized lung units. These are influenced by anatomical position within a mechanically non-homogeneous lung, gravitational dependence of position, innate fragility, and susceptibility to stress amplification. Higher intracycle peaks of total global power may occur earlier during inflation, but hypothetically, only intracycle power applied in excess of that individual unit’s individual stress threshold level has the potential to augment the damage risk by overstraining the surrounding matrix and other structural microelements that separate individual alveolar units. Under this assumption, injuring power per inflation cycle is logically a joint function of intracycle power and alveolar pressure threshold.

### Unresolved questions

In this evolving field of VILI energetics, it is not yet clear whether the ‘elastic’ power (the inflation component most directly relevant to *alveolar* energy load), or total power (which includes the power component expended in large and small airways as well as in tissue friction), is most relevant to VILI risk [12]. Clearly, flow-resistive power largely dissipates in moving gas through the endotracheal tube and through the native airways. However, it should not be dismissed as ‘not VILI relevant’ because the flow component of the inflating pressure simultaneously determines the speed with which parenchymal tissues expand [4, 5]. Not surprisingly, for the same flow pattern, separation of the total intracycle power curve from that of that of its elastic component is widened by relative increases of resistance as opposed to compliance (Additional file 2: Fig. E1).

One might reasonably question the relative places of the flow-determined maximal intracycle power above pressure threshold (*M*) and the ‘above elastic pressure threshold’ energy (*A*) within the hierarchy of damaging mechanical influences; this ranking awaits further biological evidence. Indeed, these are but two logical yet unproven energetic indicators of risk. For the individual tidal cycle, transpulmonary plateau pressure and driving pressures are perhaps pre-eminent variables that influence VILI risk and clinical outcomes [4, 12, 14, 15]. A rapid flow and high rate of parenchymal expansion might exert negligible effects unless pre-existing strain, associated driving pressures and viscoelastic retardation were also high (causing micro-strains to rise above local critical thresholds). In this context, it also seems apparent that any added importance of flow *waveform* would be conditioned by other factors. For example, flow *amplitude* is undoubtedly important; a low peak flow seems highly unlikely to influence injury hazard, whatever the profile that applies it. Another consideration is that a large capacity lung may remain unaffected by a flow rate to which a lung of smaller capacity would be vulnerable. The relevance of lung size to power concentration within aeratable tissue has been emphasized in the concept of the ‘shrinking baby lung’ and ‘VILI vortex’ [16] and may help explain why driving pressure is a better correlate of outcome than tidal volume unreferenced to compliance [15]. Along this same line, although the externally measured intracycle power levels might seem of small magnitude when related to the whole lung, certain units within the ‘baby lung’ are likely to bear the brunt of that applied power, concentrating its effect. It goes without saying that the frequency with which the threshold pressure is violated would be critical to overt VILI expression, whatever the intracycle contributors may be that breach it [2, 4, 14, 17].

### Limitations of the model

This modeling exercise comparing the intracycle power profiles delivered by standard flow waveforms was undertaken to illustrate a neglected concept of possible clinical relevance and as such is simply a starting point for more advanced work that tests its pathobiological implications. The current model does not incorporate the interactive multi-compartmental nature and non-linear expansion characteristics of the injured lungs encountered in practice. Therefore, we make no pretense regarding the quantitative precision of estimating local tissue strains. However, with the possibility for further model development and clinical applications in mind, we developed our one-compartment mathematical model to accept as inputs both the typical ventilator settings [18] and the lumped mechanical parameters for respiratory system resistance (*R*) and compliance (*C*) that clinicians commonly calculate and/or ventilators display at the bedside. This initial attempt to describe, validate and focus on the measurable intracycle power, therefore, is built upon assumptions and simplifications; consequently, its conceptual implications are subject to important limitations. Our equations modeled ‘ideal’ waveforms that abruptly rise to their initial values for pressure or flow and do not account for ‘smoothing and tapering’ by the ventilator’s gas delivery algorithms. Perhaps more importantly, the predictions for intracycle power behavior developed by these modeling equations assume an arbitrary pressure threshold that applies to the entire lung. Any such selection, however, bears only approximate relevance to regional properties that impact the local strains experienced within an actual lung, as thresholds will vary in each region. The arbitrary value we chose seemed reasonable to test, however, based on the limits for plateau and driving pressure clinically observed in today’s ‘lung protective’ strategy. We also assumed passive conditions and used airway rather than transpulmonary pressures, so that in this simplified model, contributions from the chest wall structures are ignored. Additionally, as a first step in exploring intracycle power behaviors we chose to verify the physical relevance of this mathematical model with a mechanical simulator, rather than an actual biological system. Doing so imposed a restriction on the clinical translation of that validation process, but also allowed us to directly record the simulated ‘alveolar’ pressures needed to estimate global elastic power. Finally, it must be acknowledged that higher elastic power and energy might well influence the extent of intratidal (damaging) or stabilized (beneficial) recruitment. Such recruiting effects of intracycle power are best explored in a multicompartment model, for which the current unicompartmental version could serve as a useful mathematical building block.

### Clinical implications

Although clearly in need of experimental and clinical validation, this conceptually novel intracycle power analysis is rooted in physics-determined behaviors that hold inferences for bedside practice. In certain animal models of lung disease, patterns that impose high flow transients may have greater damaging potential than those without them [19, 20]. Avoidance of high flow spikes may be a wise intervention at *deflation* onset, as well [21]. If greater attention to the flow waveform is warranted, prominent among helpful clinical measures may be to reduce minute ventilation requirements (e.g., by permissive hypercapnia, pH control and extracorporeal support) that strongly influence the required amplitudes of peak and mean inspiratory flows, as well as repetition frequency. Assuming that maximal intracycle power and energy above threshold influence VILI risk, selecting a flow waveform and an inspiratory time period that avoid high intracycle peaks of flow and power would also be indicated. For a given V_T_ and inspiratory time, the greatest and least maximal and above pressure threshold intracycle power values are generally encountered with the abruptly rising flow of pressure control and the evenly applied flow of CF, respectively. Therefore, with VILI in mind, selecting a longer ‘rise time’ (‘attack rate’) to the targeted pressure for pressure control may be prudent so as to more closely mimic the latter. With the same rationale of minimizing maximal intracycle power, extending inspiratory time potentially might offer benefit in flow-controlled modes, as well. It stands to reason that helpful measures to dampen the local effects of global intracycle power include key elements of current ‘lung protection’ strategy: using lower tidal volumes, carefully titrating PEEP to safely enlarge the ‘size’ of the aeratable compartment, and prone positioning to help even the distribution of transpulmonary pressures [22].

## Conclusion

While the varied inspiratory flow patterns ultimately deliver similar total amounts of alveolar energy during each breath, they differ profoundly regarding the potentially damaging pattern with which that energy distributes over time. Flow amplitude and waveform may be relatively neglected and modifiable determinants of VILI risk when ventilating ARDS.

## Supplementary Information

**Additional file 1: Section E1.** Mathematical Model Development. **Section E2**. Intracycle Power Definitions. **Section E3**. Comparison of Mathematical Model Predictions and Behaviors of theMechanical (Physical) Simulation for Three Prototypical Scenarios. **Section E4**. Tested Ranges for Normal, Obstructed (COPD) and ARDS ‘Virtual’ Patients. **Section E5**. Computing Intracycle Power on a Virtual Population of ARDS Patients. **Section E6**. Intracycle power above Alveolar PressureThreshold for Damage. SectionE7. Statistical Comparisons ofSupra-threshold Energy and Power for 4Flow Waveforms in 3 Disease States. 

**Additional file 2.** Marini E1 Total and Elastic Power ARDS vs COPD 

## Data Availability

Model and data are placed in public data repository (FigShare). This article has an on-line electronic supplement which can also be accessed at: https://tinyurl.com/xhx6u9md; https://figshare.com/articles/journal_contribution/Marini_Integrated_Supplement_docx/14555556; 10.6084/m9.figshare.14555556.v1.
