# Supplementary material for: Intracycle power and ventilation mode as potential contributors to ventilator-induced lung injury
Source: Intensive Care Med Exp. 2021 Nov 1;9:55. doi: 10.1186/s40635-021-00420-9 (PMC8557972; doi:10.1186/s40635-021-00420-9)
Supplement: Supplementary file 1 — Additional file 1: Section E1. Mathematical Model Development. Section E2. Intracycle Power Definitions. Section E3. Comparison of Mathematical Model Predictions and Behaviors of theMechanical (Physical) Simulation for Three Prototypical Scenarios. Section E4. Tested Ranges for Normal, Obstructed (COPD) and ARDS ‘Virtual’ Patients. Section E5. Computing Intracycle Power on a Virtual Population of ARDS Patients. Section E6. Intracycle power above Alveolar PressureThreshold for Damage. SectionE7. Statistical Comparisons ofSupra-threshold Energy and Power for 4Flow Waveforms in 3 Disease States. [file 40635_2021_420_MOESM1_ESM.docx]

On-Line Electronic Supplement

**Intracycle Power and Ventilation Mode as Potential Contributors to**

**Ventilator-Induced Lung Injury**

**Section E1: Mathematical Model Development**

A mathematical model was used to simulate ventilation into and out of a simplified one-compartment respiratory system. Total duration of each breath (*t_tot_*) was subdivided into an inspiratory phase ($0\leq t\leq t_{i}$) and an expiratory phase ($t_{i}\leq t\leq t_{tot}$). We assumed ‘lumped’ but clinically-measurable mechanical characteristics for resistance (*R*) and compliance (*C*). In mathematical terms, these assumptions translate into the equations:

$$P_{resistive}=R\cdot flow=R\left( \frac{dV}{dt} \right)$$

$$P_{elastic}=E\cdot volume=\left( \frac{1}{C} \right)V\left( t \right).$$

Here, the inflation volume $V(t)$ delivered by the ventilator is defined as zero at the beginning and the ending of the breath.

The “equations of motion” build upon this base by assuming that the applied pressure at the airway opening is balanced by pressures developing in response to resistance and elastance (inverse of compliance) as well as the residual alveolar pressure value at end-expiration, defined as$P_{residual}=P_{ex}=total PEEP, the sum of PEEP and autoPEEP$. Thus:

$$P_{resistive}+P_{elastic}+P_{residual}=P_{applied.}$$

These assumptions lead to the following mathematical model comprised of a coupled pair of initial-value problems for the unknown volume functions for inflation and expiration $V_{i}\left( t \right)\mathrm{and}V_{e}\left( t \right),\mathrm{respectively}$.

Inspiration (*i*):

$$R\frac{dV_{i}}{dt}+\frac{V_{i}}{C}+P_{ex}=P_{aw} , 0\leq t\leq t_{i}$$

$$V_{i}\left( 0 \right)=0$$

*Expiration (*e*):

$$R\frac{dV_{e}}{dt}+\frac{V_{e}}{C}+P_{ex}=PEEP,t_{i}\leq t\leq t_{tot}$$

$$V_{e}\left( t_{i} \right)=V_{i}(t_{i}{)=V}_{T.}$$

*(During expiration flow is assumed to be a negative quantity.)

Each differential equation in the mathematical model can be solved for $V_{i}\left( t \right)\mathrm{and}V_{e}(t)$. For example, in the case of pressure-controlled ventilation:

$$V_{i}\left( t \right)=CP_{ex}\left( {1-e}^{-\frac{t}{CR}} \right)+CP_{set}\left( 1-e^{-\frac{t}{CR}} \right).$$

Our simplified mathematical model emulates ventilation-related variables that result from different ventilatory patterns (flow waveforms). In addition to the sinusoidal flow pattern of normal spontaneous breathing (SF), we studied model predictions for three popular modes of ventilation: (1) constant inflation pressure ventilation (P*_set_*)$;$(2) constant flow ventilation (CF); and (3) linearly decelerating flow ventilation (DF).

**Section E2: Intracycle Power Definitions**

The total intracycle total power can be considered as the sum of its elastic PEEP and resistive components.

We define the IntraCycle *Driving* Power (${ICP}_{D}$) as the product of tidal elastic lung pressure above total PEEP and flow (Q). That is,

$${ICP}_{D}\left( t \right)=\left( \frac{1}{C} \right)V_{i}\left( t \right){\cdot Q}_{i}\left( t \right)=\left( \frac{1}{C} \right)V_{i}\left( t \right)\frac{{dV}_{i}}{dt}\left( t \right).$$

Similarly, we define the IntraCycle *Elastic* Power (*ICP_E_*), a product of the values at inspiratory time *t* for *dynamic* alveolar pressure (driving pressure plus PEEP) and flow, as:

$${ICP}_{E}\left( t \right)=\left[ \left( \frac{1}{C} \right)V_{i}\left( t \right)+P_{ex} \right]Q_{i}\left( t \right)=\left[ \left( \frac{1}{C} \right)V_{i}\left( t \right)+P_{ex} \right]\frac{{dV}_{i}}{dt}(t)$$

and the IntraCycle Total Power (*ICP_T_*) as the sum of the values at inspiratory time *t* for flow resistive power and *ICP_E_* :

$${ICP}_{T}\left( t \right)=\left[ RQ_{i}\left( t \right)+\left( \frac{1}{C} \right)V_{i}\left( t \right)+P_{ex} \right]Q_{i}\left( t \right)=\left[ R\frac{{dV}_{i}}{dt}\left( t \right)+\left( \frac{1}{C} \right)V_{i}\left( t \right)+P_{ex} \right]\frac{{dV}_{i}}{dt}\left( t \right).$$

**Section E3: Comparison of Mathematical Model Predictions and Behaviors of the Mechanical (Physical) Simulation for Three Prototypical Scenarios**

The predictions of the mathematical model were compared with experiments performed on a servo-actuated lung simulator (Ingmar ASL-5000) driven by a commercial ventilator. Ventilator settings: tidal volume, inspiratory time, frequency, and PEEP and mode of ventilation (P_set_, CF, or DF), as well as properties for patient lung mechanics (compliance and resistance), were chosen to resemble ARDS, airway obstruction (COPD), and normal lung scenarios (Table E1). Measurements of time, volume, flow, airway pressure in and out of the mechanical lung were recorded and compared against the mathematical model. In doing this, our goal was to

Determine if the mathematical model produces accurate predictions for volume and flow into the mechanical lung during inspiration, as these two quantities are used to compute values for the intracycle power functions, e.g., ${ICP}_{T}\left( t \right)=RQ_{i}\left( t \right)+\frac{V_{i}\left( t \right)}{C}+P_{ex}$, $0\leq t\leq t_{i}$.

The ventilator-lung simulator experiments were performed for a wide variety of ventilator settings and compliance-resistance combinations using 3 modes of ventilation. For example, with ventilator settings of $t_{i}=1.0 s$, $t_{tot}=3.75 s$, $V_{T}=0.35$ L, frequency = 16/min $PEEP=8.0 cmH2O$ and patient parameters of $C=0.04 L/cmH2O$, $R=8.0\frac{cmH2O}{L}/s$ with CF ventilation, we have the following comparison between the mechanical lung outputs (red dots) and mathematical model predictions (blue curve) for the volumes (L.) generated during inspiration and expiration:

The mathematical model predicted the total end-expiratory pressure (including auto-PEEP) as $P_{ex}=8.023 cmH2O$. The maximum separation ‘error’ between the simulation and model was 0.032 L and the minimum error was 5.511 x 10^-16^ L As one can readily observe, the maximum difference between the model and simulator volumes (liters) occurs during expiration. For CF and DF ventilation experiments, maximum errors were observed generally during expiration. Using DF ventilation, another experiment produced these results:

The maximum separation here is 0.039 L., again occurring during the expiratory portion of the breath.

The most noteworthy differences occurred using P_set_ ventilation. This can be seen in the following example of P_set_ ventilation.

The maximum error is 0.042 L and occurs during inflation. The central problem with P_set_ ventilation experiments lies with the airway pressure actually being delivered by the ventilator during inspiration. In constant pressure ventilation (P_set_), the mathematical model assumes that the airway pressure is constant throughout inspiration. If one examines the airway pressure developed by the ventilator during inspiration, however, it only becomes constant some brief time after inflation onset. This can be seen in the following tracing of airway pressure (cmH2O), taken from the above example.

One can see about 10-15% of the inspiratory time is spent building the airway pressure to its pre-set value. By design, the ventilator tends to generate lower than maximal pressures at the beginning of inspiration and therefore delivers lower flows (and hence, smaller volumes). This explains why the red dots of the simulator volumes undershoot the mathematical model’s predicted volume curve. The tested simulator settings and virtual patient parameters are shown in Table E2. In summary, if the mathematical model is provided accurate information about its inputs, then its predictions are very consistent with those experienced by the mechanical ventilator-simulator system.

Table E2 Conditions tested using the ventilator-simulator system

| **Exp #** | **Mode** | **Flow Waveform** | **PEEP (cm H_2_O)** | **f Rate (bp)** | **V_T_ (ml)** | | **T_I_ (sec)** | | **T_R_ (sec)** | **Flow  Ramp (%)** | **C (L/cm H_2_O)** | **R (cm H2O/L/s)** |
| --- | --- | --- | --- | --- | --- | --- | --- | --- | --- | --- | --- | --- |
| 1 | VC-CMV | Constant | 16 | 30 | | 300 | | 0.8 | 0.0 | 100 | 0.015 | 10 |
| 2 | VC-CMV | Constant | 10 | 30 | | 300 | | 0.8 | 0.0 | 100 | 0.015 | 15 |
| 3 | VC-CMV | Constant | 8 | 16 | | 400 | | 1.0 | 0.0 | 100 | 0.040 | 8 |
| 4 | VC-CMV | Constant | 8 | 16 | | 400 | | 1.0 | 0.0 | 100 | 0.060 | 8 |
| 5 | VC-CMV | Constant | 8 | 14 | | 500 | | 1.0 | 0.0 | 100 | 0.080 | 10 |
| 6 | VC-CMV | Constant | 5 | 12 | | 500 | | 1.2 | 0.0 | 100 | 0.100 | 20 |
| 7 | VC-CMV | Constant | 5 | 12 | | 500 | | 1.2 | 0.0 | 100 | 0.100 | 30 |
| 8 | VC-CMV | Descending Ramp 0% | 16 | 30 | | 300 | | 0.8 | 0.0 | 0 | 0.015 | 10 |
| 9 | VC-CMV | Descending Ramp 0% | 10 | 30 | | 300 | | 0.8 | 0.0 | 0 | 0.015 | 15 |
| 10 | VC-CMV | Descending Ramp 0% | 8 | 16 | | 400 | | 1.0 | 0.0 | 0 | 0.040 | 8 |
| 11 | VC-CMV | Descending Ramp 0% | 8 | 16 | | 400 | | 1.0 | 0.0 | 0 | 0.060 | 8 |
| 12 | VC-CMV | Descending Ramp 0% | 8 | 14 | | 500 | | 1.0 | 0.0 | 0 | 0.080 | 10 |
| 13 | VC-CMV | Descending Ramp 0% | 5 | 12 | | 500 | | 1.2 | 0.0 | 0 | 0.100 | 20 |
| 14 | VC-CMV | Descending Ramp 0% | 5 | 12 | | 500 | | 1.2 | 0.0 | 0 | 0.100 | 30 |
| 15 | VC-CMV | Descending Ramp 30% | 16 | 30 | | 300 | | 0.8 | 0.0 | 30 | 0.015 | 10 |
| 16 | VC-CMV | Descending Ramp 30% | 10 | 30 | | 300 | | 0.8 | 0.0 | 30 | 0.015 | 15 |
| 17 | VC-CMV | Descending Ramp 30% | 8 | 16 | | 400 | | 1.0 | 0.0 | 30 | 0.040 | 8 |
| 18 | VC-CMV | Descending Ramp 30% | 8 | 16 | | 400 | | 1.0 | 0.0 | 30 | 0.060 | 8 |
| 19 | VC-CMV | Descending Ramp 30% | 8 | 14 | | 500 | | 1.0 | 0.0 | 30 | 0.080 | 10 |
| 20 | VC-CMV | Descending Ramp 30% | 5 | 12 | | 500 | | 1.2 | 0.0 | 30 | 0.100 | 20 |
| 21 | VC-CMV | Descending Ramp 30% | 5 | 12 | | 500 | | 1.2 | 0.0 | 30 | 0.100 | 30 |
| 22 | VC-CMV | Descending Ramp 50% | 16 | 30 | | 300 | | 0.8 | 0.0 | 50 | 0.015 | 10 |
| 23 | VC-CMV | Descending Ramp 50% | 10 | 30 | | 300 | | 0.8 | 0.0 | 50 | 0.015 | 15 |
| 24 | VC-CMV | Descending Ramp 50% | 8 | 16 | | 400 | | 1.0 | 0.0 | 50 | 0.040 | 8 |
| 25 | VC-CMV | Descending Ramp 50% | 8 | 16 | | 400 | | 1.0 | 0.0 | 50 | 0.060 | 8 |
| 26 | VC-CMV | Descending Ramp 50% | 8 | 14 | | 500 | | 1.0 | 0.0 | 50 | 0.080 | 10 |
| 27 | VC-CMV | Descending Ramp 50% | 5 | 12 | | 500 | | 1.2 | 0.0 | 50 | 0.100 | 20 |
| 28 | VC-CMV | Descending Ramp 50% | 5 | 12 | | 500 | | 1.2 | 0.0 | 50 | 0.100 | 30 |
| 29 | PC-CMV | Exponential Decay PR0 | 16 | 30 | | 300 | | 0.8 | 0.0 | NA | 0.015 | 10 |
| 30 | PC-CMV | Exponential Decay PR0 | 10 | 30 | | 300 | | 0.8 | 0.0 | NA | 0.015 | 15 |
| 31 | PC-CMV | Exponential Decay PR0 | 8 | 16 | | 400 | | 1.0 | 0.0 | NA | 0.040 | 8 |
| 32 | PC-CMV | Exponential Decay PR0 | 8 | 16 | | 400 | | 1.0 | 0.0 | NA | 0.060 | 8 |
| 33 | PC-CMV | Exponential Decay PR0 | 8 | 14 | | 500 | | 1.0 | 0.0 | NA | 0.080 | 10 |
| 34 | PC-CMV | Exponential Decay PR0 | 5 | 12 | | 500 | | 1.2 | 0.0 | NA | 0.100 | 20 |
| 35 | PC-CMV | Exponential Decay PR0 | 5 | 12 | | 500 | | 1.2 | 0.0 | NA | 0.100 | 30 |
| 36 | PC-CMV | Exponential Decay PR0.4 | 16 | 30 | | 300 | | 0.8 | 0.4 | NA | 0.015 | 10 |
| 37 | PC-CMV | Exponential Decay PR0.4 | 10 | 30 | | 300 | | 0.8 | 0.4 | NA | 0.015 | 15 |
| 38 | PC-CMV | Exponential Decay PR0.4 | 8 | 16 | | 400 | | 1.0 | 0.4 | NA | 0.040 | 8 |
| 39 | PC-CMV | Exponential Decay PR0.4 | 8 | 16 | | 400 | | 1.0 | 0.4 | NA | 0.060 | 8 |
| 40 | PC-CMV | Exponential Decay PR0.4 | 8 | 14 | | 500 | | 1.0 | 0.4 | NA | 0.080 | 10 |
| 41 | PC-CMV | Exponential Decay PR0.4 | 5 | 12 | | 500 | | 1.2 | 0.4 | NA | 0.100 | 20 |
| 42 | PC-CMV | Exponential Decay PR0.4 | 5 | 12 | | 500 | | 1.2 | 0.4 | NA | 0.100 | 30 |

**Table E2: Ventilating settings, physiological parameters, and ventilation modes used in 42 mechanical simulator experiments.** For decelerating flow (Descending Ramp), the end-inflation flow rate was either 0%, 30% or 50% of the peak initial flow rate. Pressure Control (Pset, PC-CMV) produced an exponential decay and was tested with two approaches to the targeted value: PRO and PRO.4. These corresponded to rapid and slow ‘attack’ rates to the targeted pressure, respectively.

**Section E4 Tested Ranges for Normal, Obstructed (COPD) and ARDS ‘Virtual’ Patients**

**Table E1**  **Virtual Patients : Sample Generation and Parameter Ranges**

# 5,000 Normal Lung Samples

| **Statistic/Parameter** | Ti | Ttot | Vt | C | R | PEEP |
| --- | --- | --- | --- | --- | --- | --- |
| **Mean** | 0.898 | 2.241 | 0.524 | 0.100 | 9.984 | 5.042 |
| **SD** | 0.172 | 0.527 | 0.131 | 0.011 | 1.152 | 2.884 |
| **Max** | 1.200 | 3.390 | 0.750 | 0.120 | 11.999 | 9.992 |
| **Min** | 0.600 | 1.102 | 0.300 | 0.080 | 8.000 | 0.006 |

# 5,000 Severe Obstruction Samples

| **Statistic/Parameter** | Ti | Ttot | Vt | C | R | PEEP |
| --- | --- | --- | --- | --- | --- | --- |
| **Mean** | 1.199 | 2.397 | 0.499 | 0.085 | 26.082 | 5.018 |
| **SD** | 0.232 | 0.326 | 0.114 | 0.020 | 8.144 | 2.872 |
| **Max** | 1.600 | 3.185 | 0.700 | 0.120 | 39.997 | 9.999 |
| **Min** | 0.800 | 1.616 | 0.300 | 0.050 | 12.001 | 0.002 |

# 5,000 ARDS Samples

| **Statistic/Parameter** | Ti | Ttot | Vt | C | R | PEEP |
| --- | --- | --- | --- | --- | --- | --- |
| **Mean** | 1.105 | 2.333 | 0.368 | 0.034 | 9.970 | 11.228 |
| **SD** | 0.197 | 0.475 | 0.071 | 0.014 | 1.202 | 3.850 |
| **Max** | 1.389 | 3.281 | 0.499 | 0.060 | 11.974 | 19.764 |
| **Min** | 0.703 | 1.342 | 0.250 | 0.010 | 8.038 | 5.222 |

**Units**: Ti (s); Ttot (s); Vt (L); C (L/cmH2O); R (cmH2O/L/sec); PEEP (cmH2O)

**Section E5 Computing Intracycle Power on a Virtual Population of ARDS Patients**

In order to detail the behavior of driving, elastic, and total power for ARDS patients, a virtual population of 5,000 samples was created by randomly choosing inspiratory times, frequency, tidal volume, applied PEEP, compliance, and resistance values within certain prescribed ranges that are common to ARDS patients of varying severity. In our computations, we chose the following restrictions on these parameters: ${0.7\leq t}_{i}\leq1.4 (s)$, $0.25\leq V_{T}\leq0.5 (L)$, $5.0\leq PEEP\leq20.0 (cmH2O)$, $0.01\leq C\leq0.06 (\frac{L}{cmH2O})$, and $8.0\leq R\leq12.0 (\frac{cmH2O}{L}/s)$. Lastly, $t_{tot}=t_{i}+t_{e} (s)$ with $0.5\leq t_{e}\leq2.0$. Upon building the 5,000 ‘virtual ARDS patient’ samples, the means and standard deviations of these parameters using those restrictions were: $t_{i}=1.105\pm0.197$, $t_{tot}=2.333\pm0.475$, $V_{T}=0.368\pm0.071$, $PEEP=11.228\pm3.850$, $C=0.034\pm0.014$, and $R=9.970\pm1.202$. (**Table E1**)

We examined the intracycle power functions (${ICP}_{D}(t)$, ${ICP}_{E}(t)$, ${ICP}_{T}(t)$) on this virtual ARDS population for four modes of ventilation ($P_{set}$, $CF$, $DF$, and $SF$). Before we describe the details, we note that it can be proven mathematically for our model that

$$\int_{0}^{t_{i}} {ICP}_{D}\left( t \right)dt=\frac{1}{2C}\left( V_{T} \right)^{2}$$

and

$$\int_{0}^{t_{i}} {ICP}_{E}\left( t \right)dt=\frac{1}{2C}\left( V_{T} \right)^{2}+P_{ex}V_{T}$$

regardless which mode of ventilation is used. In other words, the areas under the intracycle driving power and intracycle elastic power functions are determined by only the tidal volume, compliance and end-expiratory pressure. However, this independence for the modes of ventilation does not hold for ${ICP}_{T}(t)$.

**Section E6 Intracycle power above Alveolar Pressure Threshold for Damage**

In the following, we examine the areas under the intracycle power curves, not on the entire inspiratory interval, but rather on the proportion of the inspiratory interval when the elastic pressure, $P_{elastic}\left( t \right)=\frac{V_{i}\left( t \right)}{C}+P_{ex}$, exceeds a specified threshold for ‘safe’ elastic pressure. Since the elastic pressure is an increasing function in time, there is an inspiratory time, $t_{ep}$, after which the elastic pressure (ep) will be above its threshold over the interval: $t_{ep}\leq t\leq t_{i}$. Using this threshold pressure, we can calculate the area under the intracycle power curve (*A*, equivalent to what may be potentially damaging elastic energy) for all four modes of ventilation.

$$A=\int_{t_{ep}}^{t_{i}} ICP\left( t \right)dt$$

The setup for energy and power is shown in the following generic figure, whose conceptual principle applies

equally well to total power and to its driving and dynamic elastic components.

*A*

The area *A* below the power curve (ICP) is a measure of the energy after the elastic pressure has crossed its threshold (at the time $t=t_{ep}$). The value of *A* varies from sample to sample, as does the threshold time ($t_{ep}$). However, there is an interesting fact about two intracycle power functions, which are the driving (A_D_) and elastic (A_E_) components of intracycle power (${ICP}_{D}and {ICP}_{E})$that meet the elastic pressure threshold criterion:

$$A_{D}=\int_{t_{ep}}^{t_{i}} {ICP}_{D}\left( t \right)dt$$

$$A_{E}=\int_{t_{ep}}^{t_{i}} {ICP}_{E}\left( t \right)dt$$

For a given sample, each of these area types is unaffected by changes in the flow profile used on that sample. This leaves the intracycle total power area function ($A_{T},$the sum of resistive area above the pressure threshold and A*_E_*) as a possible discriminator between the modes of ventilation. Therefore, we considered the area:

$$A_{T}=\int_{t_{ep}}^{t_{i}} {ICP}_{T}\left( t \right)dt$$

$A_{T}$ was individually computed for 5,000 virtual patient samples using each mode of ventilation. Using an elastic pressure threshold of 20 cmH_2_O, the following statistics for the means of $A_{T}$ were found:

- For $P_{\mathrm{set}}$ ventilation, $\bar{A}_{T}=0.513$ with a standard deviation of 0.538
- For CF ventilation, $\bar{A}_{T}=0.469$ with a standard deviation of 0.457
- For DF ventilation, $\bar{A}_{T}=0.475$ with a standard deviation of 0.471
- For SF ventilation, $\bar{A}_{T}=0.485$ with a standard deviation of 0.475.

We note that in these calculations of $A_{T}$, if the elastic pressure threshold was not reached during inspiration, then we set $A_{T}=0$ for all modes of ventilation for that sample. The mean values of $A_{T}$ for samples in which the elastic pressure threshold was not achieved, (no zero areas included) would undoubtedly be considerably higher. To determine if there are statistical differences between the modes of ventilation regarding total power, pairs of modes (e.g., CF versus SF) were tested with the Mann-Whitney Test on $A_{T}$, yielding the following results for their p-values:

- $P_{set}$ versus CF: p-value = 0.092
- $P_{set}$ versus DF: p-value = 0.120
- $P_{set}$ versus SF: p-value = 0.492
- CF versus DF: p-value = 0.855
- CF versus SF: p-value = 0.277
- DF versus SF: p-value = 0.376.

Thus, with samples having A_T_ = 0 included, the level of statistical significance (p-value = 0.05) for the area comparisons was not achieved, even though the differences between $P_{set}$ and CF or DF were very close to significance and may be achievable with a larger sample size. (This was the case with a 10,000 virtual patient sampling we conducted: $P_{set}$versus CF (p-value = 0.0092) and $P_{set}$ versus DF (p-value = 0.020).

The total power vs. time area quantity (A_T_) is a measure of the energy applied to the lung once the elastic pressure threshold is met. As such it is a macro measurement. To further understand the dynamics of the power functions, the maximum instantaneous values of intracycle power in excess of the elastic pressure criterion, $ICP(t)$, $t_{ep}\leq t\leq t_{i}$, were examined for each mode of ventilation. We introduce the notation: $M=max\left\{ ICP\left( t \right):t_{ep}\leq t\leq t_{i} \right\}$ for each type of intracycle power function, with notation of its driving (M_D_) and elastic (M_E_) components of the total (M_T_) values. As in the case of areas, if the elastic pressure threshold of 20 cmH_2_O was not achieved during inspiration, then we set $M=0$. Calculating $M$ (watts) for each mode of ventilation and each type of intracycle power function, the following mean and standard deviation statistics were found:

- For $P_{set}$ ventilation, $\bar{M}_{D}=0.551$ with a standard deviation 0.825
- For CF ventilation, $\bar{M}_{D}=0.430$ with a standard deviation 0.427
- For DF ventilation, $\bar{M}_{D}=0.322$ with a standard deviation 0.330
- For SF ventilation, $\bar{M}_{D}=0.430$ with a standard deviation 0.437
- For $P_{set}$ ventilation, $\bar{M}_{E}=1.465$ with a standard deviation 1.664
- For CF ventilation, $\bar{M}_{E}=0.814$ with a standard deviation 0.641
- For DF ventilation, $\bar{M}_{E}=0.820$ with a standard deviation 0.669
- For SF ventilation, $\bar{M}_{E}=0.966$ with a standard deviation 0.772
- For $P_{set}$ ventilation, $\bar{M}_{T}=2.595$ with a standard deviation 3.80
- For CF ventilation, $\bar{M}_{T}=0.926$ with a standard deviation 0.732
- For DF ventilation, $\bar{M}_{T}=1.053$ with a standard deviation 0.910
- For SF ventilation, $\bar{M}_{T}=1.200$ with a standard deviation 0.980.

As shown below, when checking for statistical differences between the modes of ventilation for *M_T_* $and its subcomponents$, we find (for ARDS alone) that all comparisons of modes of ventilation are significant (p-values < 10^—5^), except CF versus SF for *M_D_* (p-value = 0.537) and CF versus DF for *M_E_* (p-value = 0.224). For *M_T_*, the largest p-value was 1.8 x 10^-9^. The maximum value of the intracycle power function that exceeds threshold is therefore an excellent discriminator between modes of ventilation for the ARDS state.

**Section E7: Statistical Comparisons of Supra-threshold Energy and Power for 4 Flow Waveforms in 3 Disease States.**

We examined binary statistical comparisons between the ventilation modes for *A*, for *M*, and for their intracycle subcomponents that relate to driving and elastic power.

**Statistical Analysis****

**NORMAL Virtual Subjects**

**Driving Power Areas**

|  | Pset | Constant | Decelerating | Sinusoidal |
| --- | --- | --- | --- | --- |
| Pset | ------ | 0.999 | 0.999 | 0.999 |
| Constant | 0.999 | ------ | 0.999 | 0.999 |
| Decelerating | 0.999 | 0.999 | ----- | 0.999 |
| Sinusoidal | 0.999 | 0.999 | 0.999 | ----- |

**Elastic Power Areas**

|  | Pset | Constant | Decelerating | Sinusoidal |
| --- | --- | --- | --- | --- |
| Pset | ----- | 0.999 | 0.999 | 0.999 |
| Constant | 0.999 | ----- | 0.999 | 0.999 |
| Decelerating | 0.999 | 0.999 | ----- | 0.999 |
| Sinusoidal | 0.999 | 0.999 | 0.999 | ----- |

**Total Power Areas**

|  | Pset | Constant | Decelerating | Sinusoidal |
| --- | --- | --- | --- | --- |
| Pset | ----- | 0.987 | 0.997 | 0.988 |
| Constant | 0.987 | ----- | 0.985 | 0.999 |
| Decelerating | 0.997 | 0.985 | ----- | 0.985 |
| Sinusoidal | 0.988 | 0.999 | 0.985 | ----- |

**Driving Power Maximum**

|  | Pset | Constant | Decelerating | Sinusoidal |
| --- | --- | --- | --- | --- |
| Pset | ----- | 0.788 | 0.999 | 0.000 |
| Constant | 0.788 | ----- | 0.796 | 0.946 |
| Decelerating | 0.999 | 0.796 | ----- | 0.859 |
| Sinusoidal | 0.999 | 0.946 | 0.859 | ----- |

**Elastic Power Maximum**

|  | Pset | Constant | Decelerating | Sinusoidal |
| --- | --- | --- | --- | --- |
| Pset | ----- | 0.858 | 0.964 | 0.000 |
| Constant | 0.858 | ----- | 0.933 | 0.936 |
| Decelerating | 0.964 | 0.933 | ----- | 0.903 |
| Sinusoidal | 0.000 | 0.936 | 0.903 | ----- |

**Total Power Maximum**

|  | Pset | Constant | Decelerating | Sinusoidal |
| --- | --- | --- | --- | --- |
| Pset | ----- | 0.880 | 0.964 | 0.000 |
| Constant | 0.880 | ----- | 0.953 | 0.916 |
| Decelerating | 0.964 | 0.953 | ----- | 0.907 |
| Sinusoidal | 0.000 | 0.916 | 0.907 | ----- |

** p values by Mann-Whitney test

**Statistical Analysis****

**OBSTRUCTED Virtual Subjects**

**Driving Power Areas**

|  | Pset | Constant | Decelerating | Sinusoidal |
| --- | --- | --- | --- | --- |
| Pset | ------ | 0.999 | 1.000 | 0.999 |
| Constant | 0.999 | ------ | 0.999 | 0.999 |
| Decelerating | 1.000 | 0.999 | ----- | 0.999 |
| Sinusoidal | 0.999 | 0.999 | 0.999 | ----- |

**Elastic Power Areas**

|  | Pset | Constant | Decelerating | Sinusoidal |
| --- | --- | --- | --- | --- |
| Pset | ----- | 1.000 | 1.000 | 1.000 |
| Constant | 1.000 | ----- | 1.000 | 1.000 |
| Decelerating | 1.000 | 1.000 | ----- | 1.000 |
| Sinusoidal | 1.000 | 1.000 | 1.000 | ----- |

**Total Power Areas**

|  | Pset | Constant | Decelerating | Sinusoidal |
| --- | --- | --- | --- | --- |
| Pset | ----- | 0.987 | 0.997 | 0.988 |
| Constant | 0.987 | ----- | 0.985 | 1.000 |
| Decelerating | 0.997 | 0.985 | ----- | 0.985 |
| Sinusoidal | 0.988 | 1.000 | 0.985 | ----- |

**Driving Power Maximum**

|  | Pset | Constant | Decelerating | Sinusoidal |
| --- | --- | --- | --- | --- |
| Pset | ----- | 0.788 | 1.000 | 0.000 |
| Constant | 0.788 | ----- | 0.796 | 0.946 |
| Decelerating | 1.000 | 0.796 | ----- | 0.859 |
| Sinusoidal | 0.000 | 0.946 | 0.859 | ----- |

**Elastic Power Maximum**

|  | Pset | Constant | Decelerating | Sinusoidal |
| --- | --- | --- | --- | --- |
| Pset | ----- | 0.858 | 0.964 | 0.000 |
| Constant | 0.858 | ----- | 0.933 | 0.936 |
| Decelerating | 0.964 | 0.933 | ----- | 0.903 |
| Sinusoidal | 0.000 | 0.936 | 0.903 | ----- |

**Total Power Maximum**

|  | Pset | Constant | Decelerating | Sinusoidal |
| --- | --- | --- | --- | --- |
| Pset | ----- | 0.881 | 0.964 | 0.000 |
| Constant | 0.881 | ----- | 0.953 | 0.916 |
| Decelerating | 0.964 | 0.953 | ----- | 0.907 |
| Sinusoidal | 0.000 | 0.916 | 0.907 | ----- |

** p values by Mann-Whitney test

**Statistical Analysis****

**ARDS Virtual Subjects**

**Driving Power Areas**

|  | Pset | Constant | Decelerating | Sinusoidal |
| --- | --- | --- | --- | --- |
| Pset | ------ | 1.000 | 1.000 | 1.000 |
| Constant | 1.000 | ------ | 1.000 | 1.000 |
| Decelerating | 1.000 | 1.000 | ----- | 1.000 |
| Sinusoidal | 1.000 | 1.000 | 1.000 | ----- |

**Elastic Power Areas**

|  | Pset | Constant | Decelerating | Sinusoidal |
| --- | --- | --- | --- | --- |
| Pset | ----- | 1.000 | 1.000 | 1.000 |
| Constant | 1.000 | ----- | 1.000 | 1.000 |
| Decelerating | 1.000 | 1.000 | ----- | 1.000 |
| Sinusoidal | 1.000 | 1.000 | 1.000 | ----- |

**Total Power Areas**

|  | Pset | Constant | Decelerating | Sinusoidal |
| --- | --- | --- | --- | --- |
| Pset | ----- | 0.092 | 0.130 | 0.492 |
| Constant | 0.092 | ----- | 0.855 | 0.277 |
| Decelerating | 0.130 | 0.855 | ----- | 0.376 |
| Sinusoidal | 0.492 | 0.277 | 0.376 | ----- |

**Driving Power Maximum**

|  | Pset | Constant | Decelerating | Sinusoidal |
| --- | --- | --- | --- | --- |
| Pset | ----- | 0.000 | 8.27x10^-9^ | 0.000 |
| Constant | 0.000 | ----- | 6.58x10^-35^ | 0.537 |
| Decelerating | 8.27x10^-9^ | 6.58x10^-35^ | ----- | 6.37x10^-30^ |
| Sinusoidal | 0.000 | 0.537 | 6.37x10^-30^ | ----- |

**Elastic Power Maximum**

|  | Pset | Constant | Decelerating | Sinusoidal |
| --- | --- | --- | --- | --- |
| Pset | ----- | 1.97x10^-53^ | 1.09x10^-50^ | 0.000 |
| Constant | 1.97x10^-53^ | ----- | 0.224 | 4.55x10^-29^ |
| Decelerating | 1.09x10^-50^ | 0.224 | ----- | 3.98x10^-20^ |
| Sinusoidal | 0.000 | 4.55x10^-29^ | 3.98x10^-20^ | ----- |

**Total Power Maximum**

|  | Pset | Constant | Decelerating | Sinusoidal |
| --- | --- | --- | --- | --- |
| Pset | ----- | 3.41x10^-81^ | 6.55x10^-57^ | 0.000 |
| Constant | 3.41x10^-81^ | ----- | 1.87x10^-9^ | 1.22x10^-49^ |
| Decelerating | 6.55x10^-57^ | 1.87x10^-9^ | ----- | 1.39x10^-13^ |
| Sinusoidal | 0.000 | 1.22x10^-49^ | 1.39x10^-13^ | ----- |

** p values by Mann-Whitney test

**Table E1 Virtual Patients: Parameters and Sampling Ranges**

# 5,000 Normal Lung

**Units**: Ti (sec.); Ttot (sec.); Vt (L.); C (L/cmH2O); R (cmH2O/L/sec); PEEP (cmH2O)

| **Statistic/Parameter** | Ti | Ttot | Vt | C | R | PEEP |
| --- | --- | --- | --- | --- | --- | --- |
| **Mean** | 0.898286 | 2.24145 | 0.524453 | 0.100078 | 9.98358 | 5.04238 |
| **SD** | 0.172626 | 0.526716 | 0.130647 | 0.0113831 | 1.15246 | 2.88427 |
| **Max** | 1.19987 | 3.39309 | 0.749906 | 0.119989 | 11.9994 | 9.99176 |
| **Min** | 0.600059 | 1.10193 | 0.300301 | 0.0800088 | 8.00051 | 0.00554778 |

# 5,000 COPD

**Units**: Ti (sec.); Ttot (sec.); Vt (L.); C (L/cmH2O); R (cmH2O/L/sec); PEEP (cmH2O)

| **Statistic/Parameter** | Ti | Ttot | Vt | C | R | PEEP |
| --- | --- | --- | --- | --- | --- | --- |
| **Mean** | 1.19906 | 2.39651 | 0.498688 | 0.0848497 | 26.0815 | 5.01772 |
| **SD** | 0.231903 | 0.326325 | 0.114425 | 0.0201595 | 8.14367 | 2.87205 |
| **Max** | 1.59982 | 3.18508 | 0.699951 | 0.119997 | 39.9972 | 9.99883 |
| **Min** | 0.800219 | 1.61206 | 0.300031 | 0.0500169 | 12.0012 | 0.00182662 |

# 5,000 ARDS

**Units**: Ti (sec.); Ttot (sec.); Vt (L.); C (L/cmH2O); R (cmH2O/L/sec); PEEP (cmH2O)

| **Statistic/Parameter** | Ti | Ttot | Vt | C | R | PEEP |
| --- | --- | --- | --- | --- | --- | --- |
| **Mean** | 1.10537 | 2.33331 | 0.368201 | 0.0340092 | 9.97041 | 11.2281 |
| **SD** | 0.19703 | 0.474589 | 0.0713678 | 0.0136919 | 1.20209 | 3.84966 |
| **Max** | 1.3893 | 3.28107 | 0.498916 | 0.0599774 | 11.9748 | 19.7644 |
| **Min** | 0.7032 | 1.34288 | 0.250325 | 0.0100416 | 8.03833 | 5.22235 |

***Supplement Figure Legend***

Figure E1 Elastic and Total Intracycle power (cmH2O x L /sec) as functions of time for ARDS (a,b) and severe obstruction, exemplified by COPD (c,d). Note that for each flow profile (Pressure Control, P_set_, Constant Flow (CF), Decelerating Flow (DF), Accelerating Flow (AF) and Sinusoidal Flow (SF), the numerical separations between Total Power and Elastic Power widen as a function of resistance and compliance, which are both higher for COPD than for ARDS.
