# Supplementary material for: Intracycle power and ventilation mode as potential contributors to ventilator-induced lung injury
Source: Intensive Care Med Exp. 2021 Nov 1;9:55. doi: 10.1186/s40635-021-00420-9 (PMC8557972; doi:10.1186/s40635-021-00420-9)
Supplement: Supplementary file 2 — Additional file 2. Marini E1 Total and Elastic Power ARDS vs COPD [file 40635_2021_420_MOESM2_ESM.pdf]

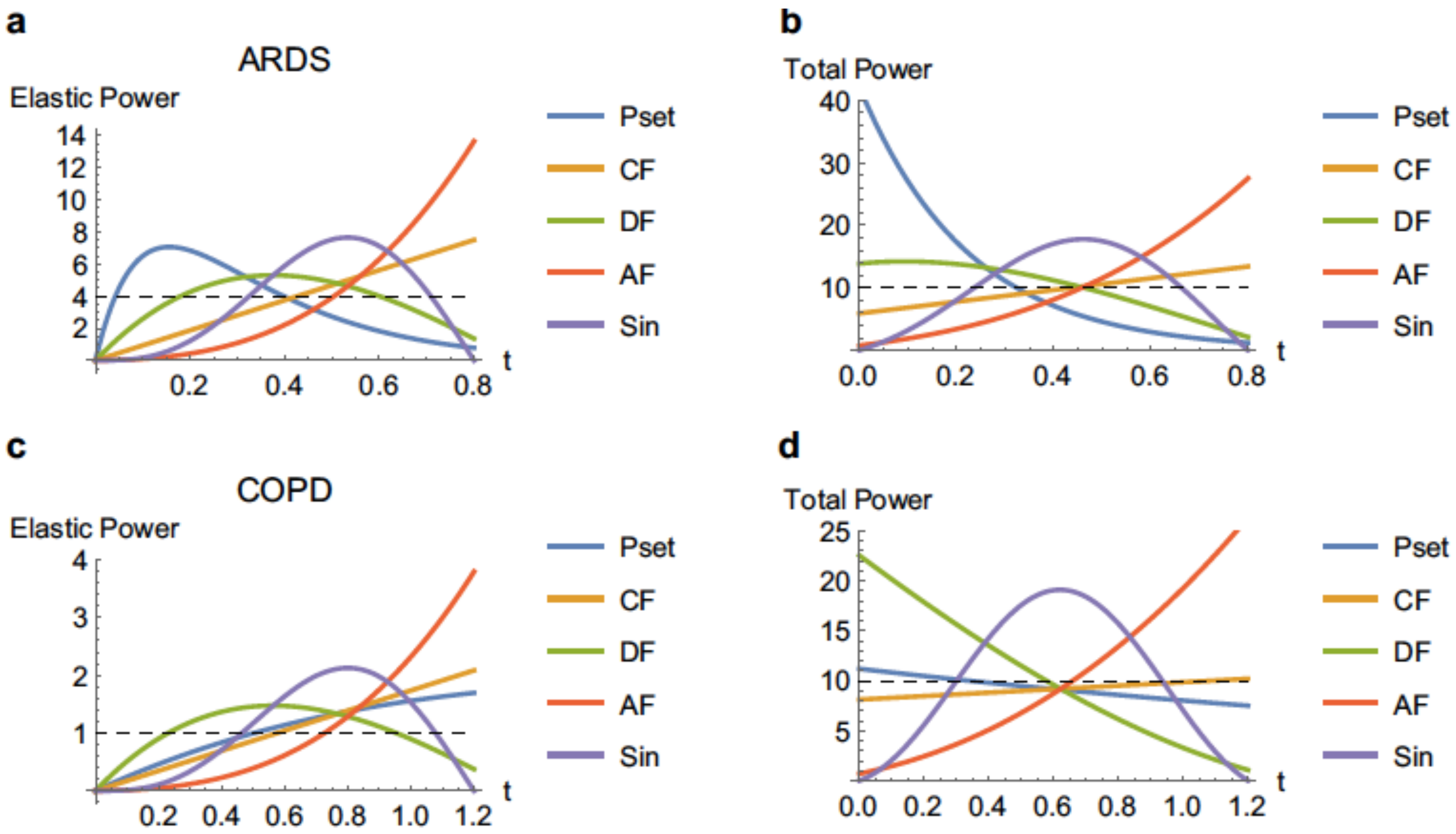

a
ARDS
Elastic Power
14
12
10
8
6
4
2
0.2
0.4
0.6
0.8
t
Pset
CF
DF
AF
Sin
b
Total Power
40
30
20
10
0.0
0.2
0.4
0.6
0.8
t
Pset
CF
DF
AF
Sin
c
COPD
Elastic Power
4
3
2
1
0.2
0.4
0.6
0.8
1.0
1.2
t
Pset
CF
DF
AF
Sin
d
Total Power
25
20
15
10
5
0.0
0.2
0.4
0.6
0.8
1.0
1.2
t
Pset
CF
DF
AF
Sin
